# Distribution of Extended-Spectrum β-Lactamase (ESBL)-Encoding Genes among Multidrug-Resistant Gram-Negative Pathogens Collected from Three Different Countries

**DOI:** 10.3390/antibiotics10030247

**Published:** 2021-03-02

**Authors:** Khaled S. M. Azab, Mohamed Ali Abdel-Rahman, Hussien H. El-Sheikh, Ehab Azab, Adil A. Gobouri, Mohamed M. S. Farag

**Affiliations:** 1 Botany and Microbiology Department, Faculty of Science, Al-Azhar University, Nasr City, Cairo 11884, Egypt; Sakr-77@hotmail.com (K.S.M.A.); HH.el-sheikh@azhar.edu.eg (H.H.E.-S.); 2 Al-Azhar Center of Fermentation Biotechnology and Applied Microbiology, Al-Azhar University, Nasr City, Cairo 11884, Egypt; 3 Department of Biotechnology, College of Science, Taif University, P.O. Box 11099, Taif 21944, Saudi Arabia; e.azab@tu.edu.sa; 4 Department of Chemistry, College of Science, Taif University, P.O. Box 11099, Taif 21944, Saudi Arabia; a.gobour@tu.edu.sa

**Keywords:** antimicrobial resistance, multidrug-resistant pathogens, resistant-genes, clinical samples, different countries

## Abstract

The incidence of Extended-spectrum β-lactamase (ESBL)-encoding genes (*bla*_CTX-M_ and *bla*_TEM_) among Gram-negative multidrug-resistant pathogens collected from three different countries was investigated. Two hundred and ninety-two clinical isolates were collected from Egypt (*n* = 90), Saudi Arabia (*n* = 162), and Sudan (*n* = 40). Based on the antimicrobial sensitivity against 20 antimicrobial agents from 11 antibiotic classes, the most resistant strains were selected and identified using the Vitek2 system and 16S rRNA gene sequence analysis. A total of 85.6% of the isolates were found to be resistant to more than three antibiotic classes. The ratios of the multidrug-resistant strains for Egypt, Saudi Arabia, and Sudan were 74.4%, 90.1%, and 97.5%, respectively. *Escherichia coli*, *Klebsiella pneumoniae*, and *Pseudomonas aeruginosa* showed inconstant resistance levels to the different classes of antibiotics. *Escherichia coli* and *Klebsiella pneumoniae* had the highest levels of resistance against macrolides followed by penicillins and cephalosporin, while *Pseudomonas aeruginosa* was most resistant to penicillins followed by classes that varied among different countries. The isolates were positive for the presence of the *bla*_CTX-M_ and *bla*_TEM_ genes. The *bla*_CTX-M_ gene was the predominant gene in all isolates (100%), while *bla*_TEM_ was detected in 66.7% of the selected isolates. This work highlights the detection of multidrug-resistant bacteria and resistant genes among different countries. We suggest that the medical authorities urgently implement antimicrobial surveillance plans and infection control policies for early detection and effective prevention of the rapid spread of these pathogens.

## 1. Introduction

Widespread use of antibiotics is often accompanied by increased bacterial resistance [1]. This can lead to the emergence of new antibiotic-resistant mechanisms that adversely affecting our ability to treat diseases. Increased antimicrobial resistance to antibiotics leads to longer treatment periods and higher health care costs. Several reports have indicated that multi-drug resistance exists in different countries such as the United States [2,3] and European countries [4]. However, resistance to microbial agents is growing very rapidly in developing countries in Africa [5] and Asia [6]. Multidrug-resistant (MDR) bacteria are non-sensitive to one or more antimicrobial agents, while extensively-drug-resistant (XDR) bacteria are non-sensitive to at least one or two agents, and pan-drug-resistant (PDR) bacteria are non-sensitive to all agents in all antimicrobial categories [7].

Extended-spectrum beta-lactamases (ESBLs) are enzymes that can hydrolyze β-lactam antibiotics. Their production, mainly depends on expression of *bla* genes, is one of the most prevalent resistance mechanisms in Gram-negative bacteria [1]. Several ESBL variants have been detected and grouped into different families including TEM, CTX-M, SHV, and OXA [8]. Among those, CTX-M and TEM are the most common ESBLs. The *bla* genes may be placed on transferable elements (plasmids or transposons) that can facilitate a horizontal spread of antibiotic resistance among different strains [3]. Besides, MDR clinical isolates are particularly worrisome to patients and clinicians and they are rapidly spreading across countries with the least resources to tackle them. Additionally, the possession of ESBLs may complicate infection control in hospital [5,6,7]. Due to the noticeable geographical differentiation of *bla* genes among ESBL-producers, examination of the prevalence of these genes among ESBL-producing strains is of great importance for clinical care and for developing optimal infection control measures in hospital [2,5].

*Escherichia coli* is the most common bacterial species in clinical laboratories, and has been implicated as playing a role in human infectious diseases [8]. *E. coli* antibiotic-resistance is very worrying because it is the most pathogenic Gram-negative bacterium. It causes urinary tract infections and both society- and hospital-acquired bacteremia and diarrhea [9]. The emergence and spread of *E. coli* resistance to several antibiotics has been reported in several regions [10].

*Klebsiella pneumoniae* is also a Gram-negative pathogen that has a large genome of plasmids and chromosomal genes. Some *Klebsiella* strains are opportunistic pathogens, infecting immunocompromised patients and causing health-care-associated infections involving pneumoniae, bloodstream infections, and urinary tract infections [11]. Other strains are hyper-virulent, infecting healthy people and causing acute infections involving endophthalmitis, pyogenic liver abscess, and meningitis [11]. Control of antimicrobial resistance in multi-drug-resistant-*Klebsiella pneumoniae* (MDR-KP) is a great challenge for clinicians. The optimal medication option for MDR-KP infections has still not been well determined. Combination therapies, including high-dose meropenem, fosfomycin, colistin, tigecycline, and aminoglycosides, are widely used, with suboptimal results [12]. The expansion of extended-spectrum β-lactamase (ESBL) production by *Klebsiella pneumoniae* (50% in some countries) is associated with high rates of mortality [13].

*Pseudomonas aeruginosa* is an opportunistic pathogen that is a major cause of morbidity and mortality in immunocompromised individuals. Elimination of *P. aeruginosa* has become increasingly difficult due to its remarkable ability to resist antibiotics [14]. *Pseudomonas aeruginosa* strains use their high levels of intrinsic and gained resistance mechanisms against most antibiotics. Also, the adaptive antibiotic resistance of *P. aeruginosa* is a recently characterized mechanism, whereby *P. aeruginosa* has developed biofilm-mediated resistance as well as forming multidrug-tolerant cells that are accountable for the recalcitrance and retrogression of infections [14].

Therefore, it is assumed that there should be continuous follow-up to reveal the extent of resistance of these pathogens to antibiotics, and results should be compared with those of preceding studies. In our previous study, we isolated different bacterial pathogens from Egypt and surrounding countries and studied their prevalence [15]. The present study was conducted to investigate the prevalence of multidrug-resistant pathogens and the antimicrobial resistance of different resistant genes in the highly resistant strains. The differences in antibiotic-resistance among strains spreading in different regions were compared.

## 2. Results

### 2.1. Study Cohort and Atrain Identification

**Figure 1:**
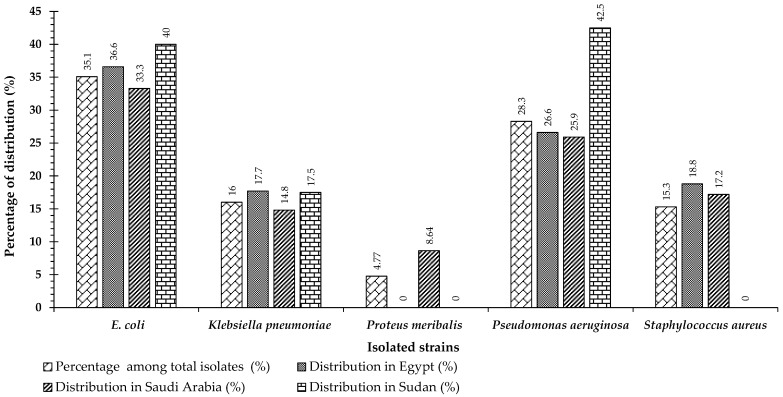
Percentages of pathogenic strains isolated from various clinical samples (collected from three different countries: Egypt, Saudi Arabia, and Sudan) among all isolates identified and the distribution of strains in each country (modified from Azab et al., [15]).

A total of 292 different clinical isolates were obtained from various discharged private laboratories in Egypt, Saudi Arabia, and Sudan from April 2015 to July 2016. Our study included 110 (37.7%) males and 182 (62.3%) females [15]. In a previous study, the most frequently isolated organism was *Escherichia coli* (*n* = 103, 35.2%), followed by *Pseudomonas aeruginosa* (*n* = 83, 28.3%), *Klebsiella pneumoniae* (*n* = 47, 16%), and *Staphylococcus aureus* (*n* = 45, 15.4%). The least frequently isolated species was *Proteus meribalis* (*n* = 14, 4.8%) [15] (Figure 1).

The distribution of isolates varied greatly among the three countries according to the sample size of each country. For *Escherichia coli*, the distribution percentage was 36.7% (*n* = 33), 33.3% (*n* = 54), and 40% (*n* = 16) for Egypt, Saudi Arabia, and Sudan, respectively. The percentages of *Klebsiella pneumoniae* were 17.8% (*n* = 16), 14.8% (*n* = 24), and 17.5% (*n* = 7), while the percentages of *Pseudomonas aeruginosa* were 26.7% (*n* = 24), 25.9% (*n* = 42), and 42.5% (*n* = 17), respectively. The distribution percentage of *Staphylococcus aureus*, (Gram-positive) was 18.9% (*n* = 17) and 17.3% (*n* = 28, 17.3%) for Egypt and Saudi Arabia, respectively. Finally, the percentage of *Proteus meribalis* was 8.6% (*n* = 14) for Saudi Arabia, but this species was not detected in the samples from Egypt and Sudan (Figure 1). Thus, *Escherichia coli*, *Pseudomonas aeruginosa*, and *Klebsiella pneumoniae* were the most repeated Gram-negative isolates in the three countries in the current investigation.

### 2.2. Antimicrobial Susceptibility Testing

The relation between antibiotic resistance and clinical specimen types was firstly investigated. Table 1 summarizes the resistance patterns of antibiotics regards the clinical specimen types. For the strains isolated from abscesses, the resistance was most commonly exhibited toward tetracycline (85%), followed by cephalosporins 2nd (82%), cephalosporins 3rd and penicillin (81%), cephalosporins 1st and macrolides (70%), quinolones (24%), and carbapenem (6%). For blood samples, the highest resistance was against macrolides (90%), followed by penicillin (82%), sulfonamide (69%), and quinolones (37%). Regarding samples isolated from the middle ear, the highest resistance was against penicillin (86%), followed by nitrofurans (77%), cephalosporins 3rd (73%), quinolones (18%), and carbapenem (9%). The Tracheal tube samples showed the highest resistance against penicillin and nitrofurans (100%), followed by cephalosporins 1st and cephalosporins 2nd (63), and tetracycline (33%). For the nasal swabs, the highest resistant were against penicillin, cephalosporins 1st and cephalosporins 2nd (100%), followed by cephalosporins 3rd, quinolones and carbapenem (67%), and finally macrolides, sulfonamides, and nitrofurans (50%). The sputum samples had the ability to resist both penicillin and the cephalosporins 2nd (88%), followed by cephalosporins 1st (86%), cephalosporins 3rd, and quinolones (75%), sulfonamide and aminoglycoside (63%), and carbapenem (50%). For throat swabs, the highest resistance was against penicillin, cephalosporins 1st and cephalosporins 2nd, macrolides, sulfonamide, and nitrofurans (100%), and aminoglycosides (33%). The results demonstrated that the samples were isolated from urethral swabs didn’t showed any resistance. For urine, the highest resistance was against penicillin (88%), followed by cephalosporins 1st (81%), tetracycline (71%), nitrofurans (29%), and carbapenem (4%). Finally, the strains isolated from vaginal swabs showed the highest resistance against macrolides (84%), followed by cephalosporins 1st (79%), penicillin (79%), and nitrofurans (31%).

**Figure 2:**
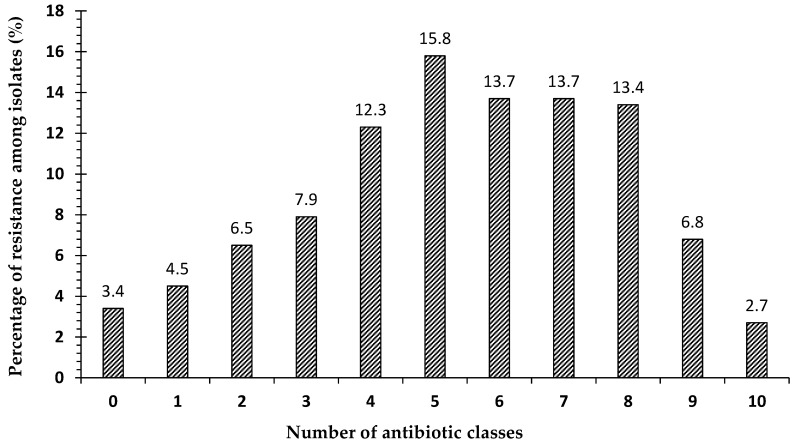
The percentage of strains resistant to antimicrobial agents from 11 antibiotic classes of the 292 bacterial strains obtained from clinical specimens: 0 means not resistant to any antibiotic group (sensitive to all groups); 1–10 indicate the numbers of classes to which the bacterial strains showed resistance; 1 indicates the percentage of strains that were resistant to only one class of antibiotic; 2 indicates the percentage of strains that were resistant to two classes of antibiotics, etc.

The patterns of the susceptibility of antibiotic classes to pathogens isolated from our clinical samples are shown in Figure 2. Most strains showed multidrug-resistance phenotypes to at least three different antimicrobial classes. In the present study, 3.4% (*n* = 10) of all strains were sensitive to all antibiotic classes, while 4.5% (*n* = 13) were resistant to one antibiotic class, and 6.5% (*n* = 19) were resistant to two antibiotic classes. Surprisingly, 85.6% (*n* = 250) of the isolates were multidrug-resistant (MDR), out of which 2.8% (*n* = 7) were extensively-drug-resistant (XDR) (Figure 2).

**Figure 3:**
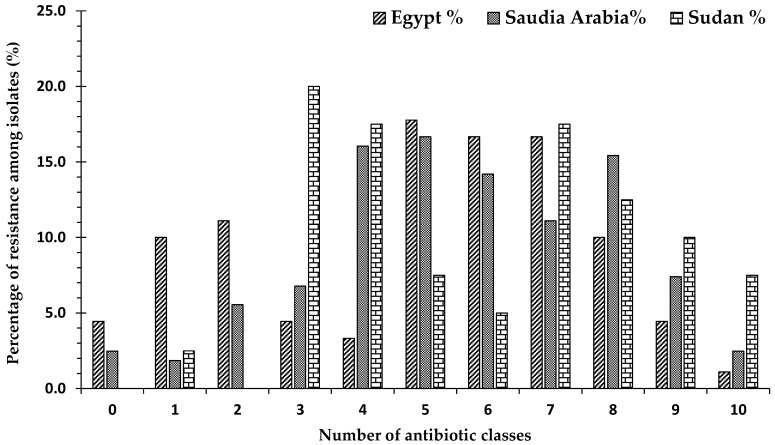
The percentages of strains resistant to antimicrobial agents from 11 antibiotic classes from all bacterial strains isolated from Egypt, Sudan, and Saudi Arabia: 0 means the percentage of strains obtained from a country that were not resistant to any antibiotic group (sensitive to all groups); numbers 1–10 indicate the numbers of classes that the bacterial strains showed resistance to; 1 indicates the percentage of strains obtained from this country that showed resistance to only one class of antibiotic; 2 indicates the percentage of strains obtained from a country that showed resistance to two classes of antibiotics amongst the 11 well known antibiotic classes, etc.

The distribution of antibiotic resistance varied among strains obtained from the three countries. Among the 90 strains obtained from Egypt, 4.4% (*n* = 4) were sensitive to all antibiotics, while 10% (*n* = 9) were resistant to one antibiotic class, and 11.1% (*n* = 10) were resistant to two antibiotic classes. A total of 74.5% (*n* = 67) strains were classified as MDR, of which 1.5% (*n* = 1) were XDR. On the other hand, of the 162 strains obtained from Saudi Arabia, only 2.5% (*n* = 4) were sensitive to all antibiotic classes, while 1.9% (*n* = 3) were resistant to one antibiotic class, and 5.6% (*n* = 9) were resistant to two classes. A total of 90% (*n* = 146) were classified as MDR, of which 2.7% (*n* = 4) were XDR. Among the 40 strains obtained from Sudan, no isolates were sensitive to all antibiotics, while 3.0% (*n* = 1) of the strains were resistant to only one class. A total of 97.5% (*n* = 39) of all strains were classified as MDR, of which 7.7% (*n* = 3) were XDR (Figure 3).

**Figure 4:**
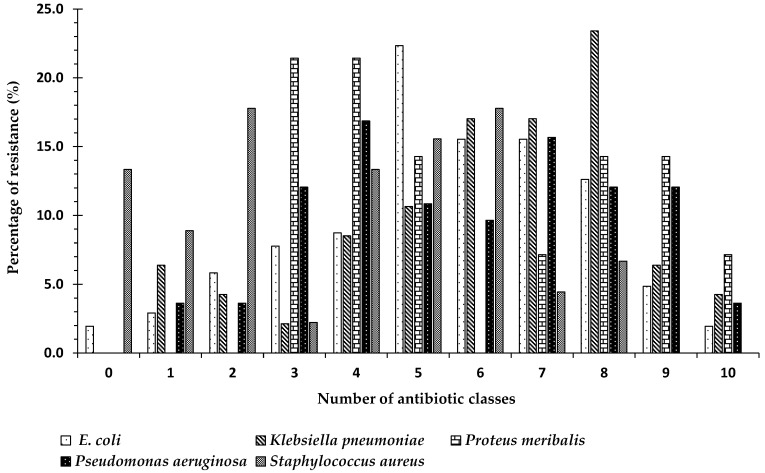
The percentage specific bacterial species that were resistant to antimicrobial agents from 11 antibiotic classes among isolated strains of the same species (obtained from 3 different countries: Egypt, Sudan, and Saudi Arabia): 0 means the percentage of strains that were not resistant to any antibiotic group (sensitive to all groups); numbers 1–10 indicate the numbers of classes to which the bacterial strain showed resistance; 1 indicates the percentage of strains that showed resistance to only one class of antibiotic; 2 indicates the percentage of strains that showed resistance to two classes of antibiotics amongst the 11 well known antibiotic classes, etc.

The diversity of drug-resistance also varied according to strain. Of the 103 *E. coli* isolates, 1.9% (*n* = 2) were sensitive to all antibiotic classes, while 2.9% (*n* = 3) and 5.8% (*n* = 6) of the *E. coli* strains were resistant to one or two classes, respectively. The percentage of *E. coli* strains that were MDR was 89.3% (*n* = 92), of which 1.9% (*n* = 1) were XDR. Of a total of 47 *K. pneumoniae* strains, 6.4% (*n* = 3) and 4.3% (*n* = 2) showed resistance to one or two classes, while no isolates were sensitive to all classes. A total of 89.3% (*n* = 42) of the *K. pneumoniae* strains were MDR, of which 4.7% (*n* = 2) were XDR. On the other hand, no *P. meribalis* strains were resistant to less than three classes, but all *P. meribalis* strains (*n* = 14) were MDR, of which 7.1% (*n* = 1) were XDR. Among the 83 *P. aeruginosa* strains, no strains were found to be sensitive to all classes, while 3.6% (*n* = 3) were resistant to either one or two classes of antibiotics. A total of 92.8% (*n* = 77) of *P. aeruginosa* strains were MDR, of which 3.9% (*n* = 3) were XDR. For the 45 obtained *S. aureus* strains, 13.3% (*n* = 6) were found to be sensitive to all classes, while 8.9% (*n* = 4) and 17.8% (*n* = 8) of the strains exhibited resistance to one or two antibiotic classes, respectively. A total of 60% (*n* = 27) were MDR, but no XDR strains were obtained (Figure 4). *Escherichia coli*, *Pseudomonas aeruginosa*, and *Klebsiella pneumoniae* were chosen for further molecular characterization and study, as they were the most frequently identified strains in the three countries under study.

### 2.3. Antibiotic Susceptibility Testing of Escherichia coli Strains

Table 2 summarizes the resistance patterns of *E. coli* strains isolated from clinical specimens of patients from the three countries. For the 33 *E. coli* strains obtained from Egypt, resistance was most commonly exhibited toward macrolides (90.9%), followed by penicillins (87.9%), cephalosporins 3rd (75.8%), cephalosporins 1st (75.0%), cephalosporins 2nd (72.7%), tetracyclines (66.7%), quinolones (57.6%), sulfonamide (48.5%), aminoglycosides (41.9%), and nitrofuran (4.5%). For the 54 *E. coli* strains isolated from Saudi Arabia, the highest level of resistance was toward macrolides (98.1%), followed by cephalosporins 1st (92.5.0%), penicillins (90.7%), tetracyclines (82.4%), sulfonamide (61.5%), cephalosporins 2nd (51.9%), aminoglycosides (48.1%), cephalosporins 3rd (44.4%), quinolones (35.2%), nitrofuran (11.1 %), and carbapenem (2.3%). Also, the level of highest resistance for the 16 *E. coli* strains obtained from Sudan was exhibited toward macrolides (100%), followed by penicillins (87.5%), cephalosporins 1st (80%), tetracyclines (80%), cephalosporins 2nd (62.5%), cephalosporins 3rd (56.3%), quinolones (50%), aminoglycosides (21.4%), and nitrofuran (6.3%).

### 2.4. Antibiotic Susceptibility of Klebsiella pneumoniae Strains

The resistance patterns of *K. pneumoniae* strains obtained from clinical specimens of patients from three countries are shown in Table 2. All 16 *K. pneumoniae* strains obtained from Egypt exhibited resistance to macrolides (100%), with 85.7% being resistance to nitrofuran, 81.3% to penicillins, 81.3% to cephalosporins 2nd, 81.3% to quinolones, 80% to cephalosporins 1st, 75% to cephalosporins 3rd, 62.5% to aminoglycosides, 56.3% to sulfonamide, 56.3% to carbapenem, and 33.3% to tetracyclines.

For the 24 *K. pneumoniae* strains obtained from Saudi Arabia, the highest number of strains were resistant to macrolides (100%), followed by penicillins (83.3%), tetracyclines (73.9%), cephalosporins 1st (70.8%), sulfonamide (54.2%), aminoglycosides (50%), cephalosporins 3rd (50%), nitrofuran (42.1%), cephalosporins 2nd (41.7%), and quinolones (33.3%). For the 7 *K. pneumoniae* strains obtained from Sudan, the highest number of strains were resistant to macrolides (100%), penicillins (100%), cephalosporins 1st (100%), cephalosporins 2nd (100%), cephalosporins 3rd (100%), quinolones (100%), followed by nitrofuran (85.7%), aminoglycosides (71.4%), sulfonamide (28.6%), and tetracyclines (28.6%)

### 2.5. Antibiotic Susceptibility of Pseudomonas aeruginosa Strains

The resistance patterns of *P. aeruginosa* isolated from clinical specimens of patients in the three countries are shown in Table 2. For the 24 *K. pneumoniae* strains obtained from Egypt, the highest number of strains were resistant to macrolides (100%), nitrofuran (100%), and penicillins (100%), followed by tetracyclines (83.3%), cephalosporins 2nd (79.2%), sulfonamide (78.3%), cephalosporins 3rd (66.7%), cephalosporins 1st (61.5%), quinolones (47.8%), aminoglycosides (41.7%), and carbapenem (25%). For the 42 *P. aeruginosa* strains obtained from Saudi Arabia, the highest number of strains were resistant to cephalosporins 1st (95.1%), followed by tetracyclines (92.7%), penicillins (90.5%), macrolides (90.5%), cephalosporins 2nd (66.8%), sulfonamide (66%), nitrofuran (54.8%), aminoglycosides (50%), cephalosporins 3rd (42.9%), quinolones (19.7%), and carbapenem (4.9%). For the 17 strains of *P. aeruginosa* isolated from Sudan, the highest number of strains were resistant to cephalosporins 2nd (100%), followed by penicillins (94.1%), cephalosporins 3rd (70.6%), cephalosporins 1st (58.9%), sulfonamide (58.8%), macrolides (35.3%), tetracyclines (29.4%), nitrofuran (18.8%), aminoglycosides (12.5%), and quinolones (11.8%).

### 2.6. Molecular Characterization of Isolates

**Figure 5:**
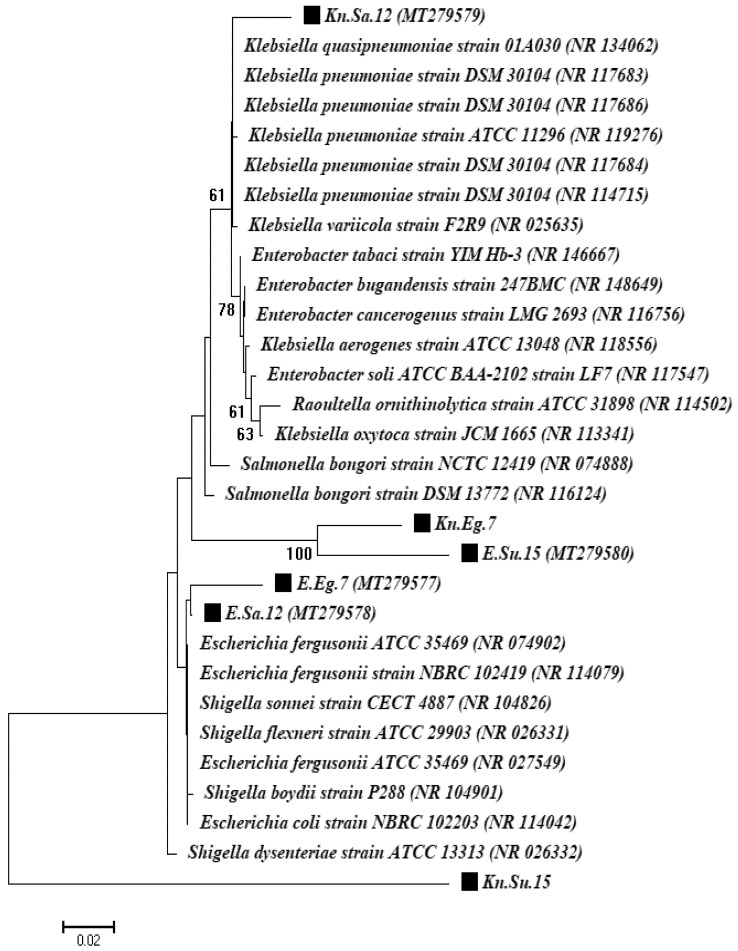
A phylogenetic tree based on 16S rRNA gene sequencing for the *Escherichia coli*, *Pseudomonas aeruginosa*, and *Klebsiella pneumoniae* strains.

Molecular characterization of the two most significant strains obtained from each country was done with 16S rRNA gene fragments. These were chosen for further molecular identification and investigation of gene resistance based on their frequency among the three countries and their high resistance to the majority of antibiotic classes. A 16S rRNA gene fragment showed that one isolate belonged to the genus *Escherichia* and was identified as the *Escherichia coli* strain and the second isolate belonged to the genus *Klebsiella* and was identified as the *Klebsiella pneumoniae* strain for all countries under study. A phylogenetic analysis based on 16S rRNA gene sequencing and alignment revealed that the two isolates were clustered with *Escherichia coli* and *Klebsiella pneumoniae* strains. The first isolates were confirmed to be *E. coli* strains (reference no. NR_114042.1) with similarity levels of 99.3%, 99.6%, and 98.6% for the Egypt, Saudi Arabia, and Sudan isolates, respectively. The second isolates were confirmed to be *K. pneumoniae* strains (reference strain nos. NR_114715.1, NR_117686.1, and NR_119276.1) with similarity levels of 98.8%, 96.7%, and 96.9% for Egypt, Saudi Arabia, and Sudan isolates, respectively (Figure 5).

### 2.7. Frequency of Antibiotic Resistance

**Figure 6:**
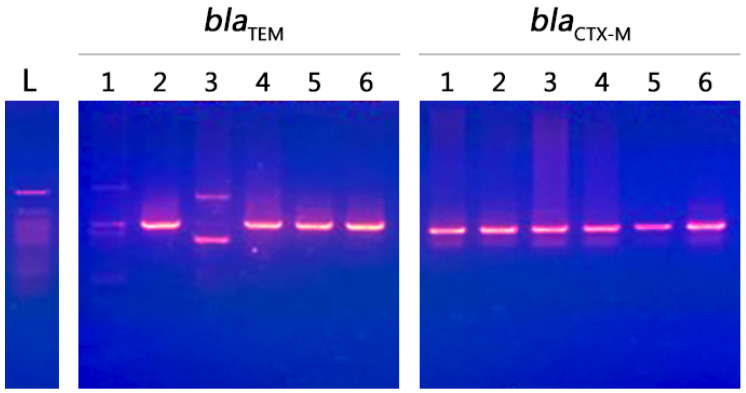
The presence of *bla*_CTX-M_ and *bla*_TEM_ genes. *bla*_CTX-M_ genes in multidrug-resistant isolates obtained from three different countries are shown. The DNA ladder is 50 bp and the *bla*_TEM_ gene (size 600 bp) was found in most of the selected isolates (*n* = 4); it was not detected in *E. coli* and *K. pneumoniae* strains obtained from Saudi Arabia and Egypt, respectively. The *bla*_CTX-M_ gene (size 500 bp) was found in all selected isolates (*n* = 6). The *bla*_CTX-M_ gene was detected in *E. coli* and *K. pneumoniae* obtained from the three countries included in this study; 1, 2, and 5 are codes for the selected *E coli* strains from Saudi Arabia, Egypt, and Sudan, respectively; 3, 4, and 6 are codes for the selected *K. pneumoniae* strains from Egypt, Saudi Arabia, and Sudan, respectively.

Our results showed that all selected strains were resistant to macrolides, nitrofuran, penicillins, tetracyclines, cephalosporins 1st, cephalosporins 2nd, cephalosporins 3rd, quinolones, aminoglycosides, sulfonamide, and carbapenem. These strains were positive for the presence of *bla*_CTX-M_ and *bla*_TEM_ genes. The predominant gene in this study was *bla*_CTX-M_ (size 500 bp), which was found in all selected isolates (*n* = 6, 100%). The *bla*_TEM_ gene (size 600 bp) was found in most of the selected isolates (*n* = 4, 66.7%). *bla*_CTX-M_ was detected in *E. coli* and *K. pneumoniae* obtained from the three countries included in this study, while the *bla*_TEM_ gene was not detected in the *E. coli* and *K. pneumoniae* strains obtained from Saudi Arabia and Egypt, respectively (Figure 6).

## 3. Discussion

Investigating the prevalence of antimicrobial resistance rates is important for creating empirical treatment strategies and evaluating the existing guidelines. The frequency and number of types of infection caused by Gram-negative bacteria have increased dramatically in the past few decades, with disparities between reports from different institutions and countries [16]. Infection with multidrug-resistant Gram-negative bacteria is a global problem that is correlated with increased morbidity and mortality rates [17]. Bacteria can obtain antibiotic resistance through mutational changes or by gaining resistance genes through horizontal gene transfer [18]. The prevalence of antimicrobial resistance is increasing worldwide, particularly in developing countries, because of greater access to antibiotic drugs. This increase in microbial drug resistance is mainly caused by the use of antimicrobials in humans and other animals [19].

In our previous study, a total of 292 different clinical isolates obtained from various discharged private laboratories from countries including Egypt, Saudi Arabia, and Sudan were evaluated to determine the pathogen distribution. In that study, the most frequently isolated organism was *Escherichia coli*, followed by *Pseudomonas aeruginosa*, *Klebsiella pneumoniae*, and *Staphylococcus aureus*. The least frequently isolated organism was *Proteus mirabilis* [15]. Our reported incidences were used to compare the prevalence of pathogens in different countries in the same region and to study multidrug resistance.

In the current study, we evaluated antimicrobial susceptibility patterns, and the results showed that a small percentage (3.4%) of the total isolated organisms were sensitive to all classes of antibiotics and a small percentage (2.7%) were resistant to ten classes of a total of eleven classes (extensively drug-resistant). The most common level of resistance was resistance to five classes, followed by resistance to six, seven, and eight classes, respectively. We found that 85.6% of all isolates were resistant to three or more (≥3) antibiotic classes, so the majority of isolates were multidrug-resistant (MDR). The highest frequency of multidrug-resistant isolates was detected in Sudan with (97.5%) followed by Saudi Arabia (90.1%) and Egypt (74.4%), while 50.3% of all strains were resistant to six antibiotic classes or more (≥6): Percentages of 48.9%, 50.6% and 49.9% for Egypt, Saudi Arabia, and Sudan, respectively. This high frequency is comparable with those found in other reports. The highest percentage of organisms that are sensitive to all antibiotic classes was found in Egypt, and the lowest rate of resistance was found in Saudi Arabia, while no organisms were detected to be sensitive to all groups in Sudan. In contrast, Sudan had the highest percentage of strains that were resistant to nine and ten groups, followed by Saudi Arabia and Egypt.

Our findings are in agreement with previous studies conducted in Sudan, which showed that *E. coli*, *K. pneumoniae*, *P. aeruginosa*, *S. aureus*, and *Proteus* spp. are the most commonly encountered organisms, and most of these strains have been found to be highly resistant to several antibiotic classes [20]. Inconsistent with another study, we showed that most isolates were resistant to many antibiotics, and Gram-negative isolates were resistant to 67% of the examined antibiotics [21]. This is in contrast to earlier publications that demonstrated that the most common pathogen isolated from different clinical specimens was *K. pneumoniae* followed by *P. aeruginosa* and then *E. coli*, and about 22.3% were resistant to three or more classes of antibiotics [22].

Our findings agree with previous studies conducted in Egypt, which showed that 93.6% of the isolated Gram-negative bacteria were multidrug-resistant [23]. Other studies have reported that 71.1% of isolates studied exhibited multidrug-resistance [24], and 91% of all *E. coli* strains were resistant to three or more antibiotics [25]. In another study conducted on bacterial isolates in drinking water in Cairo, the majority of the tested strains (62.4 to 98%) exhibited multiple antibiotic resistance [26]. Previous data from two major hospitals in Makkah, KSA showed that 24.6% of *E. coli* strains, 34.4% of *K. pneumoniae* strains, and 52.7% of *P. aeruginosa* strains were resistant to ceftazidime (cephalosporin 3rd generation) [27]. They documented the increasing prevalence of extended-spectrum beta-lactamase (ESBL) producing isolates from Saudi Arabia; in some institutes, 29% of *E. coli* strains and 65% of *K. pneumoniae* strains were ESBL [28].

In the present study, the species with the highest number strains that exhibited resistance to ≥3 antibiotic classes (multidrug-resistant) was *P. aeruginosa* (92.6%), followed by *K. pneumoniae* (89.3%), and *E. coli* (89.2%). The *E. coli* strains obtained in our study showed inconsistent resistance levels to all classes of antibiotics except carbapenem between countries. The most common pathogen for *E. coli* to be resistant to was macrolides (96.3% of all strains) followed by penicillins (88.7%), cephalosporin 1st (82.5%), tetracyclines (76.4%), cephalosporin 2nd (62.4%) and cephalosporin 3rd (58.8%), while low levels of resistance were found for nitrofuran (7.3%) and carbapenem (2.3%).

Our findings are inconsistent with the results of previous studies conducted in Sudan, which showed that multidrug-resistant *E. coli* strains were frequently resistant to macrolides [29], penicillins [21,30,31], cephalosporins [21,22,31,32], and tetracyclines [21,31], while they were less frequently resistant to quinolones, aminoglycosides [22,31], and carbapenems [22]. Other studies recorded complete sensitivity toward carbapenems [30,32], and others recorded frequent resistance against carbapenems [33]. In contrast, another study in Saudi Arabia showed that a high percentage of *E. coli* strains were resistant to penicillin [34,35], followed by cephalosporins [34], macrolides, and aminoglycosides [35], whereas few strains were resistant to aminoglycosides and nitrofuran [34], and all were sensitive to carbapenems [34]. Other studies in Egypt reported that a high percentage of *E. coli* strains were highly resistant to beta-lactams [36], followed by penicillins [37,38], cephalosporines [38], macrolides, tetracyclines, and quinolones [37,38], and aminoglycosides [37]. The lowest level of resistance was exhibited against nitrofuran and carbapenems, and aminoglycosides [38]. In contrast, another study showed that all isolated *E. coli* strains were sensitive to carbapenem [36].

In the current study, *K. pneumoniae* strains showed inconstant resistance levels to all classes of antibiotics except carbapenem. *K. pneumoniae* strains were most commonly resistant to macrolides followed by penicillins and then cephalosporin 1st, but the results for other groups were variable. In Saudi Arabia and Sudan, the lowest number of strains were resistant to carbapenem, but in Egypt, resistance was least commonly observed for tetracycline. However, this is in contrast to earlier publications from Sudan that demonstrated that a high number of *K. pneumoniae* strains were resistant to penicillins [21], followed by cephalosporines [21,22,30], nitrofuran, carbapenem, and quinolones [21]. Resistance was least commonly found for carbapenem, quinolones [22], aminoglycosides [21,22], and tetracyclines [21]. Other reported studies showed complete sensitivity toward carbapenems [30,39]. Studies in Saudi Arabia showed a high degree of resistance of *K. pneumoniae* to penicillins and tetracyclines [39,40], cephalosporins, carbapenems, aminoglycosides, and nitrofuran [40]. Resistance was least commonly found toward aminoglycosides [39,40], carbapenems [40], and quinolones [39]. Previous studies in Egypt showed that *K. pneumoniae* were highly resistant to cephalosporins [41,42,43], followed by penicillins, carbapenem, quinolones, and aminoglycosides [41,42].

In the present study, *P. aeruginosa* strains showed inconstancy in their resistance level to all classes of antibiotics, except carbapenem. The pathogen that strains were most commonly resistant to varied from macrolides to cephalosporin among countries, and the pathogens associated with the highest level of sensitivity were carbapenem and tetracycline. Studies in Sudan have shown that *P. aeruginosa* strains are most frequently resistant toward penicillins [21], followed by cephalosporines [22,44], quinolones, nitrofuran, and tetracyclines [21], and aminoglycosides [44]. Resistance was least commonly found for carbapenem, aminoglycosides, and quinolones [22]. Other studies in Saud Arabia have shown frequent resistance of *P. aeruginosa* to penicillins and carbapenems [45,46] and a low frequency of resistance toward aminoglycosides and cephalosporins 4th generation [45,46]. In contrast with our findings, other studies in Egypt showed that *P. aeruginosa* most frequently exhibited resistance against carbapenems [47,48,49], followed by extended-spectrum beta-lactamases [49], penicillins, cephalosporins, macrolides, and tetracyclines [47]. Resistance was least frequently exhibited toward carbapenems [50]. Other studies showed widespread occurrence of Gram-negative bacteria with resistance to beta-lactam and carbapenem in Middle Eastern hospitals [51].

The frequencies and types of infections caused by extended-spectrum β-lactamase (ESBL) producing pathogens have increased dramatically in the past few decades with disparities between different regions and countries. Since the beginning of the new millennium, Gram-negative bacteria have become the most commonly isolated ESBL-producing bacteria worldwide with *bla*_CTX-M_ ESBLs being the most frequently isolated type. Thus, we evaluated the prevalence of *bla*_CTX-M_ and *bla*_TEM_ resistant gene traits from clinical specimens within different countries. Resistant genes were characterized at the molecular level. In the current study, the selected isolates were found to be positive for the presence of *bla*_CTX-M_ and *bla*_TEM_ genes. The predominant gene identified in this study was *bla*_CTX-M_, which was found in all selected isolates (100%). The *bla*_TEM_ gene was found in most isolates (66.7%), the *bla*_CTX-M_ was detected in *E. coli* and *K.pneumoniae* for all countries included in our study, while the *bla*_TEM_ gene was not detected in *E. coli* and *K. pneumoniae* strains isolated from Saudi Arabia and Egypt, respectively. Our findings are in agreement with previous studies that showed that *bla*_CTX-M_ is more prevalent among tested strains of *E. coli*, *K. pneumoniae*, and *P. mirabilis* as compared with *bla*_TEM_ [52]. A study in Egypt revealed 46 *E. coli* isolates and showed that all extended-spectrum beta-lactamase (ESBL) producers were positive for *bla*_CTX-M_ and *bla*_TEM_ genes [53]. Another study carried out on *K. pneumoniae* showed that *bla*_CTX-M_ and *bla*_TEM_ genes were detected in 52.8% and 53.3% of ESBL isolates, respectively [54,55]. In another study, *bla*_CTX-M_ and *bla*_TEM_ genes were detected in *K. pneumoniae* more frequently than *E. coli*, and *bla*_TEM_ genes were detected more frequently than *bla*_CTX-M_ [38]. In agreement with our findings, studies conducted in different regions of Sudan have shown that *E. coli* is the most predominant isolate with the *bla*_CTX-M_ genotype, followed by *K. pneumoniae* [56,57]. In another study, the *bla*_TEM_ gene was detected in 55.1% of *E. coli* strains and 58% of *K. pneumoniae* strains, but the *bla*_CTX-M_ gene was detected in 71.4% of *E. coli* strains and 68.4% of *K. pneumoniae* strains [54]. Other studies conducted in Saudi Arabia detected both genes in a large number of *K. pneumoniae* strains isolated from two different hospitals. The results showed that the isolates contained the *bla*_CTX-M_ and *bla*_TEM_ genes [53,58].

Our results present alarming evidence of a severe spread of ESBL genes in the studied countries, especially the epidemiological *bla*_CTX-M_ and *bla*_TEM_ genes. There is potential for the dissemination of MDR strains in different regions. ESBLs are plasmid-mediated β-lactamases that have been recognized for their ability to hydrolyze different generations of cephalosporins (oxyimino-cephalosporin) and other antibiotic classes. These enzymes are repressed by β-lactamase inhibitors such as clavulanic acid and tazobactam. Public health efforts should focus on the correct use of antibiotics to limit their dissemination. In addition, further investigation of the molecular epidemiology of ESBLs in various clinical specimens is needed to obtain a better database of ESBL-producing pathogens present in different countries.

## 4. Methods

### 4.1. Study Design and Specimen Collection

A total of 292 clinical specimens were collected over the period from April 2015 to July 2016. Bacterial isolates of Gram-negative pathogens were obtained from various discharged private laboratories; the clinical specimens were collected from three countries (Egypt, Saudi Arabia, and Sudan). Of the 292 samples, 90 (30.8%) were from Egypt, 162 (55.5%) were from Saudi Arabia, and 40 (13.7%) were from Sudan. Bacterial strains were collected from different clinical specimens (urine (*n* = 168), vaginal swab (*n* = 43), ear swab (*n* = 22), blood (*n* = 19), abscess (*n* = 17), endotracheal tube (*n* = 8), sputum (*n* = 8), throat swab (*n* = 3), nasal swab (*n* = 3), and urethral swab (*n* = 1)). The study protocol was approved by the ethics committee, and the research followed the principles of the Declaration of Helsinki. All specimens were collected aseptically and transported to the microbiology laboratory, where they were immediately processed according to the standard microbiological procedures [59].

### 4.2. Isolation and Identification of Isolates

The samples were cultured by the streaking on blood agar [60], cysteine-lactose-electrolyte-deficient agar [61], chocolate agar [62], MacConkey agar [63], and Thayer martin agar [64], and then incubated at a 37 °C for 24 hours. The bacterial colonies were purified and identified according to the colonies’ morphology. The pathogenic isolates were identified basely by physiological and different biochemical tests [65].

Vitek2 system was used for the identification of isolates (bioMerieux, Vitek2 Compact System Version: 07.01, GN card), HCWW- Reference Lab. Microbiology Lab. GN card is based on 64 biochemical methods and newly developed substrates that measuring carbon source utilization, enzymatic activities, and resistance [66].

### 4.3. Antimicrobial Susceptibility Testing 

The antimicrobial sensitivity of pathogenic isolates to different antimicrobial agents was determined using the Kirby–Bauer disc diffusion method; each isolate was seeded on Mueller–Hinton agar following the Clinical and Laboratory Standards Institute [67] guidelines. Several antimicrobial discs representing different classes of antimicrobial agents were included in the presented study. These discs including the following: Penicillin combination, which was Amoxicillin-clavulanic acid (30 μg) and Ampicillin sulbactam (20 μg), Cephalosporin 1st generation which represented Cephalexin (30 μg), Cephalosporin 2nd generation which involved Cefuroxime (30 μg) and Cefaclor (30 μg), Cephalosporin 3rd generation containing Cefixime (5 μg), Cefotaxime (30 μg), Ceftriaxone (30 μg) and Ceftazidime (30 μg), Macrolides including Erythromycin (15 μg), Clarithromycin (15 μg) and Azithromycin (15 μg), Gentamicin (10 μg) as Aminoglycosides, Tetracycline /Doxycycline (30 μg), Quinolones/Ciprofloxacin (5 μg), Levofloxacin (5 μg), and Ofloxacin (5 μg), Sulfonamides/Trimethoprim/sulfamethoxazole (Co-trimoxazole) (25 μg), Nitrofurantoin (300 μg), and imipenem (10 μg). All antibiotic discs were produced by Oxoid (Oxoid, UK). A colony from each isolate was grown in Mueller Hinton broth (Oxoid, UK) at 37 °C. The discs of antibiotics were applied using fine-end forceps, and plates were incubated at 37 °C for 24 hrs. Inhibition zones were measured and interpreted as sensitive (S), intermediate (I), or resistant (R) according to Clinical and Laboratory Standards criteria [68]. Isolates that show resistance to at least three different classes of antimicrobial agents were considered as MDR.

### 4.4. Molecular Identification of Bacterial Isolates

Molecular identification was performed by the extraction of genomic DNA using the modified method as previously described [69,70]. The 16S rRNA gene fragments were analyzed using forward and reverse universal primers as 27f (5 GAGTTTGATCACTGGCTCAG-3) and 1492r (5-TACGGCTACCTTGTTACGACTT-3). Genomic DNA was used as a template for polymerase chain reaction (PCR). The PCR mixture contained the folloing: PCR buffer (1×), dNTP (0.25 mM), MgCl_2_ (0.5 mM), Taq DNA polymerase [QIAGEN] (2.5 U), primers (0.5 μM of each), and genomic DNA (1 μg of). The PCR was conducted in DNA Engine Thermal Cycler (PTC-200, Bio-Rad, Hercules, CA, USA) at the following conditions: Hot starting performed at 94 °C (3 min), then 30 cycles of 94 °C (0.5 min), 55 °C (0.5 min), and 72 °C (1 min). The extension was performed at 72 °C for 10 min. The PCR products were commercially sequenced at Sigma Company (Cairo, Egypt) using an ABI 3730xl DNA sequencer. Through BLASTN, The sequences were compared with sequences available at GenBank database. Multiple sequence alignments were then performed by ClustalX 1.8 software package and the phylogenetic tree was established using a neighbor-joining method of Kimura 2-parameter model to calculate genetic distance as the transitional and transversional substitution rates using MEGA (version 6.1) software. The level of confidence for each branch at 1000 repeats was tested by bootstrap analysis. Detection of chimeras (>3% diverged from the closest sequences) was conducted using Uchime2_NCBI tool. 16S rRNA gene sequences of all strains have been deposited in NCBI Gene Bank using Bankit program and assigned with accession numbers MT279577- MT279580.

### 4.5. Molecular Screening of Resistant Genes

Bacterial template DNA was prepared using the boiled lysate method for molecular screening of two different resistance genes (*bla*_TEM_ and *bla*_CTX-M_ genes). For *bla*_TEM_ gene, the forward primer was *bla*_TEM_-F (5′-GCTCACCCAGAAACGCTGGT-3′), and the reverse primer was *bla*_TEM_-R (5′-CCATCTGGCCCCAGTGCTGC-3′). For *bla*_CTX-M_ gene, the forward primer was *bla*_CTXM-_ F (5′-SCSATGTGCAGYACCA GTAA-3′), and reverse primer was *bla*_CTX-M_-R (5′-ACCAGAAYVAGCGGBGC-3′). The oligonucleotide primers *bla*_TEM_ was designed based on the nucleotide sequence of *bla*_TEM_ gene reported in the National Center for Biotechnology Information (NCBI) Gene Bank database, while *bla*_CTX-M_ primers were synthesized according to a previously published protocol [71]. The PCR mixture (25 μ) contained 1 μL of each primer (10 *p*mol/μL), 12.5 μL of 2× Taq PCR RED Master Mix (Ampliqon, Denmark), 3 μL of template DNA, and 7.5 μL of ultra pure water. The PCR conditions for amplification of the *bla*_TEM_ and *bla*_CTX-M_ genes were selected based on the properties of the primers and were as follows, initial denaturation for 5 min at 94 °C, 35 cycles of denaturation for 45 s at 94 °C, annealing for 30 s at 54 °C, and extension for 60 s at 72 °C with a final extension for 5 min at 72 °C; and initial denaturation for 3 min at 94 °C, 35 cycles of denaturation for 60 s at 94 °C, annealing for 30 s at 58 °C, and extension for 60 s at 72 °C with a final extension for 10 min at 72 °C, respectively. The PCR products were subjected to agarose gel (1.2%) electrophoresis in in borate buffer (TBE, Tris-Borate-EDTA) at 80 V for 2 h and was visualized by a UV illumination after ethidium bromide (0.5 μg/mL) staining. The molecular weight was estimated by comparing with 50 bp DNA Ladder (InVitrogen) marker.

### 4.6. Statistical Analysis

Collected data was analyzed using Statistical Package for Social Sciences (SPSS; Version 23) software to calculate frequencies and percentages for all categories of organisms and resistance of isolated organisms against different antibiotics among the three countries.

## 5. Conclusions

This work highlights the detection of multidrug-resistant bacteria and resistant genes in different countries. These bacteria and genes compromise the effectiveness of different antibiotic classes. Thus, implementation of antimicrobial surveillance plans and infection control policies by medical authorities to ensure early detection and effectively halt the rapid spread of these pathogens is required.

## Figures and Tables

**Table 1:** Summarizes the percentage antibiotic resistance patterns for strains isolated from various clinical specimen types.

	Percentage of Resistance to Various Antibiotic Classes:										
Clinical Specimen	Penicillins	Cephalosporins 1st	Cephalosporins 2nd	Cephalosporins 3rd	Macrolides	Aminoglycosides	Tetracyclines	Quinolones	Sulfonamides	Nitrofurans	Carbapenems
Abscess	81	70	82	81	70	57	85	24	67	57	6
Blood	82	46	50	54	90	59	55	37	69	0	64
Ear Swab	86	68	55	73	67	64	50	18	59	77	9
Endotracheal tube	100	63	63	57	0	38	33	38	57	100	38
Nasal Swap	100	100	100	67	50	33	100	67	50	50	67
Sputum	88	86	88	75	-	63	67	75	63	-	50
Throat Swap	100	100	100	67	100	33	50	0	100	100	0
Urethral	0	0	0	0	0	0	0	0	0	0	0
Urine	88	81	61	54	91	45	71	43	59	29	4
Vaginal Swap	79	80	47	49	84	32	70	35	49	31	0

**Table 2:** Summary of the patterns of resistance to antibiotic classes for *Escherichia coli*, *Pseudomonas aeruginosa*, and *Klebsiella pneumoniae* strains obtained from three different countries.

Resistance to:	*E. coli* Strains (%)			*Klebsiella pneumoniae* Strains (%)			*Pseudomonas aeruginosa* Strains (%)		
	Egypt	Sudan	Saudi Arabia	Egypt	Sudan	Saudi Arabia	Egypt	Sudan	Saudi Arabia
Penicillins	87.9	87.5	90.7	81.3	100.0	83.3	100.0	94.1	90.5
Cephalosporins 1st	75.0	80.0	92.5	80.0	100.0	70.8	61.5	58.9	95.1
Cephalosporins 2nd	72.7	62.5	51.9	81.3	100.0	41.7	79.2	100.0	66.8
Cephalosporins 3rd	75.8	56.3	44.4	75.0	100.0	50.0	66.7	70.6	42.9
Macrolides	90.9	100.0	98.1	100.0	100.0	100.0	100.0	35.3	90.5
Aminoglycosides	41.9	21.4	48.1	62.5	71.4	50.0	41.7	12.5	50.0
Tetracyclines	66.7	80.0	82.4	33.3	28.6	73.9	83.3	29.4	92.7
Quinolones	57.6	50.0	35.2	81.3	100.0	33.3	47.8	11.8	16.7
Sulfonamide	48.5	66.7	61.5	56.3	28.6	54.2	78.3	58.8	66.0
Nitrofuran	4.5	6.3	11.1	85.7	85.7	42.1	100.0	18.8	54.8
Carbapenem	0	0	2.3	56.3	0	0.0	25.0	0	4.9

## Data Availability

The datasets used and analyzed in the current study are available from the corre-sponding author upon reasonable request.

## Abbreviations

*bla*: β-lactamase; bp: Base pair; DNA: Deoxyribonucleic acid; ESBL: Extended-spectrum β-lactamase; F: Forward; GN: Gram-negative; HCWW: Holding Company for water and waste Water; KSA: Kingdom of Saudi Arabia; MDR: Multidrug-resistant; MDR-KB: multi-drug-resistant-*Klebsiella pneumoniae*; *n*: Number; NCBI: National Center for Biotechnology Information; PCR: Polymerase chain reaction; PDR: Pandrug resistant; R: Reverse; rRNA: Ribosomal ribonucleic acid; SPSS: Statistical Package for Social Sciences; XDR: Extensively drug-resistant.

## References

[1] Davies J., Davies D. (2010). Origins and evolution of antibiotic resistance. Microbiol. Mol. Biol. Rev..

[2] Sahm D.F., Thornsberry C., Mayfield D.C., Jones M.E., Karlowsky J.A. (2001). Multidrug-resistant urinary tract isolates of *Escherichia coli*: Prevalence and patient demographics in the United States in 2000. Antimicrob. Agents Chemother..

[3] Karlowsky J.A., Jones M.E., Draghi D.C., Thornsberry C., Sahm D.F., Volturo G.A. (2004). Prevalence and antimicrobial susceptibilities of bacteria isolated from blood cultures of hospitalized patients in the United States in 2002. Ann. Clin. Microbiol. Antimicrob..

[4] Van De Sande-Bruinsma N., Grundmann H., Verloo D., Tiemersma E., Monen J., Goossens H., Ferech M., European Antimicrobial Resistance Surveillance System Group, European Surveillance of Antimicrobial Consumption Project Group (2008). Antimicrobial drug use and resistance in Europe. Emerg. Infect. Dis..

[5] Okeke I.N., Aboderin O.A., Byarugaba D.K., Ojo K.K., Opintan J.A. (2007). Growing problem of multidrug-resistant enteric pathogens in Africa. Emerg. Infect. Dis..

[6] Chen Y.-H., Hsueh P.-R., Badal R.E., Hawser S.P., Hoban D.J., Bouchillon S.K., Ni Y., Paterson D.L. (2011). Antimicrobial susceptibility profiles of aerobic and facultative Gram-negative bacilli isolated from patients with intra-abdominal infections in the Asia-Pacific region according to currently established susceptibility interpretive criteria. J. Infect..

[7] Magiorakos A.-P., Srinivasan A., Carey R.B., Carmeli Y., Falagas M.E., Giske C.G., Harbarth S., Hindler J.F., Kahlmeter G., Olsson-Liljequist B. (2012). Multidrug-resistant, extensively drug-resistant and pandrug-resistant bacteria: An international expert proposal for interim standard definitions for acquired resistance. Clin. Microbiol. Infect..

[8] Koneman E.W., Allen S.D., Janda W., Schreckenberger P., Winn W. (1997). Diagnostic microbiology. The Nonfermentative Gram-Negative Bacilli.

[9] Coleman B.L., Louie M., Salvadori M.I., McEwen S.A., Neumann N., Sibley K., Irwin R.J., Jamieson F.B., Daignault D., Majury A. (2013). Contamination of Canadian private drinking water sources with antimicrobial resistant *Escherichia coli*. Water Res..

[10] Nys S., Okeke I.N., Kariuki S., Dinant G.J., Driessen C., Stobberingh E.E. (2004). Antibiotic resistance of faecal *Escherichia coli* from healthy volunteers from eight developing countries. J. Antimicrob. Chemother..

[11] Martin R.M., Bachman M.A. (2018). Colonization, infection, and the accessory genome of *Klebsiella pneumoniae*. Front. Cell. Infect. Microbiol..

[12] Bassetti M., Righi E., Carnelutti A., Graziano E., Russo A. (2018). Multidrug-resistant *Klebsiella pneumoniae*: Challenges for treatment, prevention and infection control. Expert Rev. Anti-Infect. Ther..

[13] Paterson D.L., Ko W.-C., Von Gottberg A., Mohapatra S., Casellas J.M., Goossens H., Mulazimoglu L., Trenholme G., Klugman K.P., Bonomo R.A. (2004). Antibiotic Therapy for *Klebsiella pneumoniae* Bacteremia: Implications of production of extended-spectrum β-Lactamases. Clin. Infect. Dis..

[14] Pang Z., Raudonis R., Glick B.R., Lin T.-J., Cheng Z. (2019). Antibiotic resistance in *Pseudomonas aeruginosa*: Mechanisms and alternative therapeutic strategies. Biotechnol. Adv..

[15] Azab K.S.M., Abdel-Rahman M.A., Farag M.M.S., El-Sheikh H.H. (2020). Identification and distribution of pathogenic bacteria in clinical specimens within Egypt, Saudi Arabia, and Sudan. Al-Azhar Bull. Sci..

[16] Chong Y., Shimoda S., Shimono N. (2018). Current epidemiology, genetic evolution and clinical impact of extended-spectrum β-lactamase-producing *Escherichia coli* and *Klebsiella pneumoniae*. Infect. Genet. Evol..

[17] Higgins P., Wisplinghoff H., Krut O., Seifert H. (2007). A PCR-based method to differentiate between *Acinetobacter baumannii* and *Acinetobacter genomic* species 13TU. Clin. Microbiol. Infect..

[18] Munita J.M., Arias C.A. (2016). Mechanisms of antibiotic resistance. Microbiol. Spectr..

[19] Abd El-Hamid M.I., Bendary M.M., Merwad A.M.A., Elsohaby I., Ghaith D.M., Alshareef W.A. (2019). What is behind phylogenetic analysis of hospital-, community- and livestock-associated methicillin-resistant *Staphylococcus aureus*?. Transbound. Emerg. Dis..

[20] Abass A.M., Ahmed M.E., Ibrahim I.G., Yahia S.A. (2017). Bacterial resistance to antibiotics: Current situation in Sudan. J. Adv. Microbiol..

[21] Saeed A., Hamid S.A., Bayoumi M., Shanan S., Alouffi S., Alharbi S.A., Alshammari F.D., Abd H. (2017). Elevated antibiotic resistance of Sudanese urinary tract infection bacteria. EXCLI J..

[22] Elbadawi H.S., Elhag K.M., Mahgoub E., Altayb H.N., Hamid M.M.A. (2019). Antimicrobial resistance surveillance among Gram-negative bacterial isolates from patients in hospitals in Khartoum State, Sudan. F1000Research.

[23] Khalifa H.O., Soliman A.M., Ahmed A.M., Shimamoto T., Nariya H., Matsumoto T., Shimamoto T. (2019). High prevalence of antimicrobial resistance in Gram-negative bacteria isolated from clinical settings in Egypt: Recalling for judicious use of conventional antimicrobials in developing nations. Microb. Drug Resist..

[24] Shehab El-Din E.M.R., El-Sokkary M.M.A., Bassiouny M.R., Hassan R. (2015). Epidemiology of neonatal sepsis and implicated pathogens: A study from Egypt. BioMed Res. Int..

[25] Ali M.M.M., Ahmed S.F., Klena J.D., Mohamed Z.K., Moussa T., Ghenghesh K.S. (2014). Enteroaggregative *Escherichia coli* in diarrheic children in Egypt: Molecular characterization and antimicrobial susceptibility. J. Infect. Dev. Ctries..

[26] El-Zanfaly H.T., Kassim E.-S.A.-A., Badr-Eldin S.M. (1987). Incidence of antibiotic resistant bacteria in drinking water in Cairo. Water Air Soil Pollut..

[27] Zowawi H.M. (2016). Antimicrobial resistance in Saudi Arabia. An urgent call for an immediate action. Saudi Med. J..

[28] Zowawi H.M., Balkhy H.H., Walsh T.R., Paterson D.L. (2013). β-Lactamase production in key Gram-negative pathogen isolates from the Arabian Peninsula. Clin. Microbiol. Rev..

[29] Elmofti H.A., Almofti Y., Abuelhassan N.N., Omer N.N. (2019). Identification and antibiotic resistance patterns of *Escherichia coli* isolated from broilers farms in Bahri locality/Sudan. Acta Sci. Nutr. Health.

[30] Mekki A., Hassan A., Eldin D., Elsayed M. (2010). Extended spectrum beta lactamases among multi drug resistant *Escherichia coli* and Klebsiella species causing urinary tract infections in Khartoum. J. Bacteriol. Res..

[31] Ibrahim M., Bilal N., Hamid M. (2013). Increased multi-drug resistant *Escherichia coli* from hospitals in Khartoum state, Sudan. Afr. Health Sci..

[32] Ahmed O.B., Omar A.O., Asghar A.H., Elhassan M.M. (2013). Increasing prevalence of ESBL-producing Enterobacteriaceae in Sudan community patients with UTIs. Egypt. Acad. J. Biol. Sci. G Microbiol..

[33] Satir S., Elkhalifa A., Ali M., Rahim A., Elhussein A., Elkhidir I., Enan K. (2016). Detection of carbepenem resistance genes among selected Gram negative bacteria isolated from patients in-Khartoum state, Sudan. Clin. Microbiol..

[34] Yasir M., Ajlan A.M., Shakil S., Jiman-Fatani A.A., Almasaudi S.B., Farman M., Baazeem Z.M., Baabdullah R., Alawi M., Al-Abdullah N. (2018). Molecular characterization, antimicrobial resistance and clinico-bioinformatics approaches to address the problem of extended-spectrum β-lactamase-producing *Escherichia coli* in western Saudi Arabia. Sci. Rep..

[35] Hemeg H.A. (2018). Molecular characterization of antibiotic resistant *Escherichia coli* isolates recovered from food samples and outpatient Clinics, KSA. Saudi J. Biol. Sci..

[36] Aly M.E., Essam T.M., Amin M.A. (2012). Antibiotic resistance profile of *E. coli* strains isolated from clinical specimens and food samples in Egypt. Int. J. Microbiol. Res..

[37] Amer M.M., Mekky H.M., Amer A.M., Fedawy H.S. (2018). Antimicrobial resistance genes in pathogenic *Escherichia coli* isolated from diseased broiler chickens in Egypt and their relationship with the phenotypic resistance characteristics. Vet. World.

[38] Mohammed H., Elsadek Fakhr A., Al Johery S.a.E., Hassanein W.A.G. (2016). Spread of TEM, VIM, SHV, and CTX-M β-lactamases in imipenem-resistant Gram-negative bacilli isolated from Egyptian hospitals. Int. J. Microbiol..

[39] Ahmad S., Al-Juaid N.F., Alenzi F.Q., Mattar E.H., Bakheet O.E.-S. (2009). Prevalence, antibiotic susceptibility pattern and production of extended-spectrum beta-lactamases amongst clinical isolates of *Klebsiella pneumoniae* at Armed Forces Hospital in Saudi Arabia. J. Coll. Physicians Surg. Pak..

[40] Azim N.S.A., Nofal M.Y., Alharbi M.A., Al-Zaban M.I., Somily A.M. (2019). Molecular-diversity, prevalence and antibiotic susceptibility of pathogenic *Klebsiella pneumoniae* under Saudi Condition. Pak. J. Biol. Sci..

[41] Shawky S.M., Abdallah A., El Kholy M. (2015). Antimicrobial activity of Colistin and Tigecycline against carbapenem resistant *Klebsiella pneumoniae* clinical isolates in Alexandria, Egypt. Int. J. Curr. Microbiol. Appl. Sci..

[42] Wasfi R., Elkhatib W.F., Ashour H.M. (2016). Molecular typing and virulence analysis of multidrug resistant *Klebsiella pneumoniae* clinical isolates recovered from Egyptian hospitals. Sci. Rep..

[43] El-Badawy M.F., Tawakol W.M., El-Far S.W., Maghrabi I.A., Al-Ghamdi S.A., Mansy M.S., Ashour M.S., Shohayeb M.M. (2017). Molecular identification of Aminoglycoside-modifying enzymes and plasmid-mediated Quinolone resistance genes among *Klebsiella pneumoniae* clinical isolates recovered from Egyptian patients. Int. J. Microbiol..

[44] Khosravi A.D., Taee S., Dezfuli A.A., Meghdadi H., Shafie F. (2019). Investigation of the prevalence of genes conferring resistance to carbapenems in *Pseudomonas aeruginosa* isolates from burn patients. Infect. Drug Resist..

[45] Alkeshan Y. (2014). Antimicrobial Resistance Pattern of *Pseudomonas aeruginosa* in Regional Tertiary Care Hospitals of Saudi Arabia. IOSR J. Dent. Med. Sci..

[46] Khan M.A., Faiz A. (2016). Antimicrobial resistance patterns of *Pseudomonas aeruginosa* in tertiary care hospitals of Makkah and Jeddah. Ann. Saudi Med..

[47] Gad G.F., El-Domany R.A., Zaki S., Ashour H.M. (2007). Characterization of *Pseudomonas aeruginosa* isolated from clinical and environmental samples in Minia, Egypt: Prevalence, antibiogram and resistance mechanisms. J. Antimicrob. Chemother..

[48] Elshafiee E.A., Nader S.M., Dorgham S.M., Hamza D.A. (2019). Carbapenem-resistant *Pseudomonas aeruginosa* originating from farm animals and people in Egypt. J. Vet. Res..

[49] Farhan S.M., Ibrahim R.A., Mahran K.M., Hetta H.F., El-Baky R.M.A. (2019). Antimicrobial resistance pattern and molecular genetic distribution of metallo-β-lactamases producing *Pseudomonas aeruginosa* isolated from hospitals in Minia, Egypt. Infect. Drug Resist..

[50] Helmy O.M., Kashef M.T. (2017). Different phenotypic and molecular mechanisms associated with multidrug resistance in Gram-negative clinical isolates from Egypt. Infect. Drug Resist..

[51] Dandachi I., Chaddad A., Hanna J., Matta J., Daoud Z. (2019). Understanding the epidemiology of multi-drug resistant Gram-negative bacilli in the Middle East using a one health approach. Front. Microbiol..

[52] Ojdana D., Sacha P., Wieczorek P., Czaban S., Michalska-Falkowska A., Jaworowska J., Jurczak A., Poniatowski B., Tryniszewska E. (2014). The occurrence of *bla*_CTX-M_, *bla*_SHV_, and *bla*_TEM_ genes in extended-spectrum β-Lactamase-positive strains of *Klebsiella pneumoniae*, *Escherichia coli*, and *Proteus mirabilis* in Poland. J. Antibiot..

[53] Al-Agamy M.H.M., Ashour M.S.E.-D., Wiegand I. (2006). First description of CTX-M β-lactamase-producing clinical *Escherichia coli* isolates from Egypt. Int. J. Antimicrob. Agents.

[54] Ahmed O., Omar A., Asghar A., El hassan M. (2013). Prevalence of TEM, SHV and CTX-M genes in *Escherichia coli* and *Klebsiella* spp urinary isolates from Sudan with confirmed ESBL phenotype. Life Sci. J..

[55] Elkhateeb H., Abdel-Mongy M., Othman A. (2018). In vitro Detection of Antibacterial Activity of Glycyrrhizic Acid Nanoparticle against ESBL Producing *Klebsiella pneumoniae* Strains. Egypt. J. Microbiol..

[56] Osman A.E.M.A.E., Hashim S.O., Musa M.A., Tahir O.M. (2017). Detection of CTX-M, TEM and SHV Genes in Gram negative bacteria isolated from nosocomial patients at Port Sudan Teaching Hospital. Eur. J. Clin. Biomed. Sci..

[57] Altayb H.N., Siddig M., El Amin N.M., Maowia A.I.H., Mukhtar M. (2018). Molecular Characterization of CTX-M ESBLs among Pathogenic Enterobacteriaceae isolated from different regions in Sudan. Glob. Adv. Res. J. Microbiol..

[58] El Hassan M., Ozbazk H.A., Hemeg H.A., Ahmed A. (2016). Dissemination of CTX-M extended-spectrum β-lactamases (ESBLs) among *Escherichia coli* and *Klebsiella pneumoniae* in Al-Madenah Al-Monawwarah region, Saudi Arabia. Int. J. Clin. Exp. Med..

[59] Winn W.C., Allen S.D., Janda W.M., Koneman E.W., Procop G., Schreckenberger P., Woods G.L. (2005). Color Atlas and Textbook of Diagnostic Microbiology.

[60] Spector W.S. (1955). Handbook of Toxicology.

[61] Sandys G.H. (1960). A new method of preventing swarming of *Proteus* sp. with a description of a new medium suitable for use in routine laboratory practice. J. Med. Lab. Technol..

[62] McLeod J.W., Wheatley B., Phelon H.V. (1927). On Some of the Unexplained Difficulties met with in cultivating the *Gonococcus*: The part played by the amino-acids. Br. J. Exp. Pathol..

[63] MacConkey A.T. (2009). Note on some Cases of Food-poisoning. Epidemiology and Infection.

[64] Thayer J.D., Martin J.E. (1964). A selective medium for the cultivation of *N. gonorrhoeae* and *N. meningitidis*. Public Health Rep..

[65] Sutherland C. (2008). District Laboratory Practice in Tropical Countries 2nd edition, Part 1. Monica Cheesbrough 454 pp Price £50 ISBN 0521676304 Cambridge: CUP, 2005. Trop. Dr..

[66] Pincus D.H., Zbinden R., Bosshard P.P., Bottger E.C. (2007). New Vitek 2 Colorimetric GN Card for Identification of Gram-Negative Nonfermentative Bacilli. J. Clin. Microbiol..

[67] Andrews J.M. (2001). Determination of minimum inhibitory concentrations. J. Antimicrob. Chemother..

[68] Clinical and Laboratory Standards Institute (2015). Performance Standards for Antimicrobial Susceptibility Testing: 25th Informational Supplemen; CLSI Document M100–S25.

[69] Miller D.N., Bryant J.E., Madsen E.L., Ghiorse W.C. (1999). Evaluation and optimization of DNA extraction and purification procedures for soil and sediment samples. Appl. Environ. Microbiol..

[70] Jenkins C., Ling C.L., Ciesielczuk H.L., Lockwood J., Hopkins S., McHugh T.D., Gillespie S.H., Kibbler C.C. (2012). Detection and identification of bacteria in clinical samples by 16S rRNA gene sequencing: Comparison of two different approaches in clinical practice. J. Med. Microbiol..

[71] Sundsfjord A., Simonsen G.S., Haldorsen B.C., Haaheim H., Hjelmevoll S.-O., Littauer P., Dahl K.H. (2004). Genetic methods for detection of antimicrobial resistance. Acta Pathol. Microbiol. Immunol. Scand..

